# Rigidity-Induced Controlled Aggregation of Binary Colloids

**DOI:** 10.1021/acsomega.3c04909

**Published:** 2023-09-27

**Authors:** Zakiya Shireen, Tine Curk, Christian Brandl, Sujin B Babu

**Affiliations:** † Department of Mechanical Engineering, Faculty of Engineering and Information Technology, University of Melbourne, 3010 Parkville, Victoria Australia; ‡ Department of Materials Science and Engineering, Whiting School of Engineering, Johns Hopkins University, Baltimore, Maryland 21218, United States; § Out of Equilibrium Group, Department of Physics, Indian Institute of Technology Delhi, 110016 New Delhi, India

## Abstract

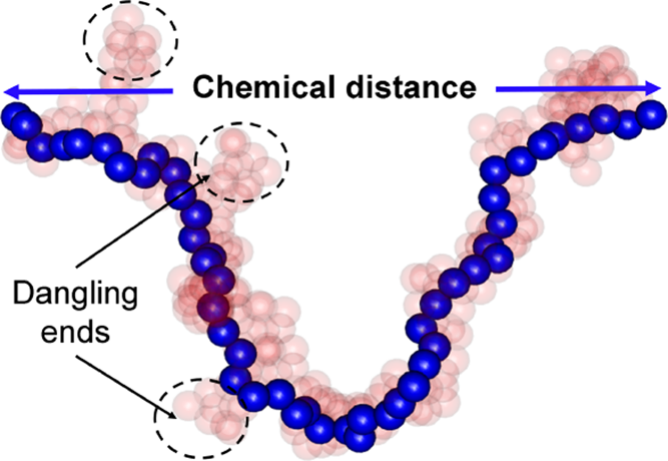

Here, we report the proof-of-concept for controlled aggregation in a binary colloidal system. The binary systems are studied by varying bond flexibility of only one species, while the other species’ bonds remain fully flexible. By establishing the underlying relation between gelation and bond rigidity, we demonstrate how the interplay among bond flexibility, critical concentration, and packing volume fraction influenced the aggregation kinetics. Our result shows that rigidity in bonds increases the critical concentration for gels to be formed in the binary mixture. Furthermore, the average number of bonded neighbor analyses reveal the influence of bond rigidity both above and below critical concentrations and show that variation in bond flexibility in only one species alters the kinetics of aggregation of both species. This finding improves our understanding of colloidal aggregation in soft and biological systems.

## Introduction

Colloidal aggregation mechanisms have been the focus of active research in various fields ranging from drug discovery^1^ to clinical medicine,^2^ protein assemblies,^3^ food science,^4^ and soft matter.^5,6^ Due to its interdisciplinary application, the aggregation of colloidal particles is a subject of interest for tuning the finite-size clusters with tailored morphology for bottom-up fabrication of energy conversion, catalysis, and sensing devices.^7,8^ To obtain complex micro and nanostructures, the morphology of the colloidal aggregates could be actively controlled using critical Casimir forces.^9,10^ Furthermore, in a recent study, phase behavior and the structure of colloidal assemblies are controlled using the flexibility of linkers in colloid–linker mixtures.^11^

The self-assembly or aggregation phenomenon has also been widely studied to understand the gelation process. Gelation occurs through dynamic arrest of the interpenetrating network via phase separation. Most importantly, the gelation phenomenon is known to be of great significance in biological systems. In living cells, biomolecule aggregation (or gelation) causes neurodegenerative diseases or cellular response to external environments.^12−14^ In drug discovery research, it is widely reported that many drugs self-assemble into colloidal aggregates, which leads to artifacts in early drug discovery.^15,16^ These artifacts include false positives and negatives in biochemical and cellular assays, respectively.^17^

Experimental insight into the aggregation process primarily relies on scattering methods, such as X-ray or neutron scattering. These techniques yield average values of microscopic quantities such as the aggregate size.^18−20^ Additionally, the rheological measures provide the mechanical signatures of aggregation and gelation of colloidal materials. A comprehensive study of DNA-coated colloidal self-assembly has enriched the understanding of the macroscopic behavior of materials via nano- and microscopic phenomena. The collective behavior of these colloids shows that reducing the number of interacting sites results in a reduced gas–liquid coexistence region with decreased critical parameters.^21^ More recently, a direct measure of the interaction potential between DNA-coated colloids by total internal reflection microscopy (TIRM) predicted the melting and binding behavior. A quantitative tuning of DNA-mediated binding systems is demonstrated to achieve high control over macroscopic material properties like the melting temperature.^22^

Contrary to general consensus that gels form by the dynamical arrest of phase separation, the latest experimental study identified the new route of gelation in a dilute colloidal suspension where the percolation occurs “after” the formation of mechanically stable rigid clusters. This also leads to the identification of two separate types of gels, stressed and stress-free gel, which is solely determined by the volume fraction with short-range interactions.^5^ In general, the aggregation process typically follows the pathway of multiple stages like preaggregation, in which thermally denatured monomers form small clusters or oligomers, followed by the association of these small clusters or aggregates into self-similar structures that gel or form a system spanning clusters.^20^ Usually, these steps occur in parallel, making the aggregation process challenging to unwind. Despite its integrative significance, a general mechanism of colloidal aggregation remains elusive.

In this work, we present a computational model of binary colloids to understand the aggregation process through bond rigidity. As a proof-of-concept, we model the binary system with irreversible bond formation in both species of the colloidal mixture. The irreversible system mimics the recent experimentally realized protein microparticle aggregation process.^4^ By tuning the bond flexibility in only one species of the binary system, we demonstrate that the kinetics of colloidal aggregation can be altered. The change in kinetics is observed only in one species and in both species depending upon the combination of packing volume fraction and critical concentration. So far, the aggregation of small molecules in drugs and percolation or gelation phenomenon has often been understood only in terms of critical concentration. Here, we demonstrate that the critical concentration of aggregators is not a singular parameter for tuning the gelation or controlling the aggregation of colloidal aggregates. We report that the interplay between critical concentration, bond flexibility, and packing volume fraction dictates control over aggregation. In addition, we also establish that the bond flexibility has a significant effect on the aggregation process below the critical concentration. In this work, we focus on the kinetics of both species and the significance of bond rigidity in the gelation process. Additionally, the critical concentration in this work is not the same as that of the critical aggregation concentration (CAC) or critical micelle concentration (CMC).^23^ We determined the critical concentration for the appearance of bigel in a binary colloidal mixture without directly associating the term with CAC or CMC.

## Model and Methods

In our previous work, we studied the kinetics, structure, and dynamics of binary colloids using Brownian Cluster Dynamics (BCD).^24,25^ To focus on the effects of bond flexibility on aggregation kinetics and local structures, we introduce a bond flexibility (*p*_flex_) parameter into the BCD model. The new flexibility parameter allows us to vary the bond flexibility from completely flexible to fully rigid bonds in one of the species (A) of a binary (A–B) system. For example, if *p*_flex_ = 0, the bonds between monomers are rigid, whereas at *p*_flex_ = 1, the bonds between A particles are fully flexible; i.e., the particles move freely within the interaction range while bonded. We vary *p*_flex_ between 0 and 1, for instance, at *p*_flex_ = 0.1, monomers are allowed to move once in every 10 movement steps; similarly, for *p*_flex_ = 0.5, the monomers are free to move five times for every 10 movement steps while bonded. Bonds are always fully flexible for B species clusters.

The initial configuration consists of *N*_tot_ randomly distributed hard spheres of unit diameter (σ = 1) and unit mass in a three-dimensional (3D) box of size *L*, modeling a well-mixed solution. The total volume fraction of the model system is given by eq 1
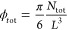
1

**Figure 1:**
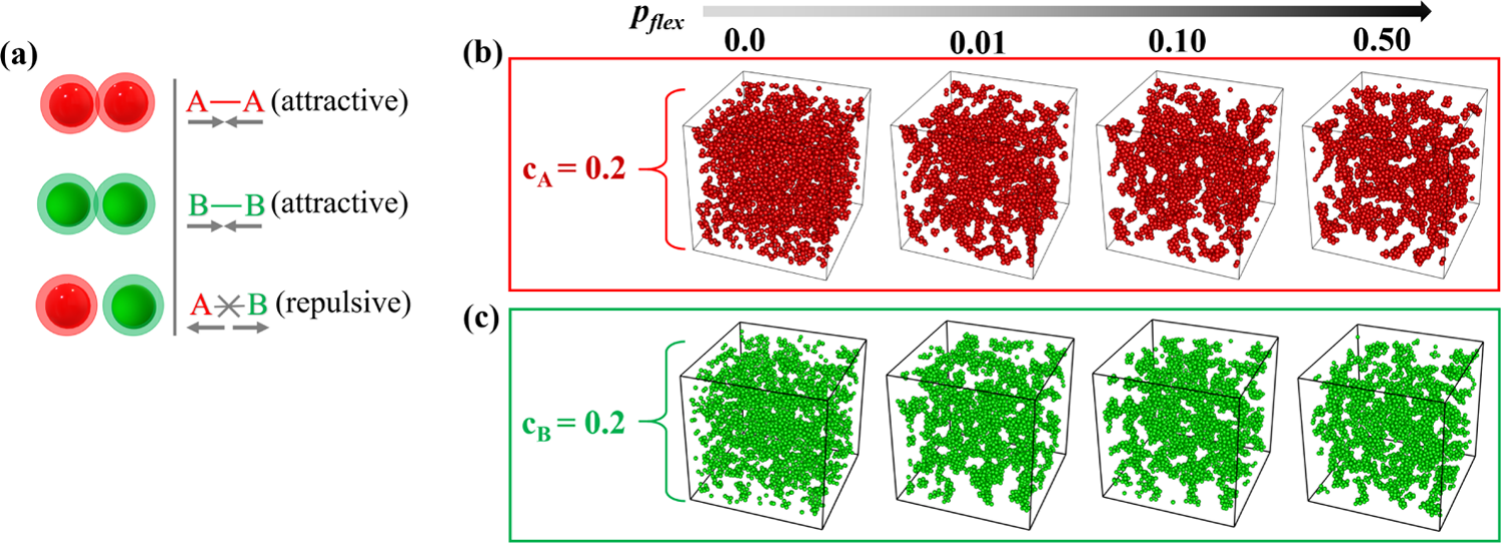
(a) Schematic of intra- (A–A, B–B) and interspecies (A–B) interaction. (b) Snapshots of the binary system in the steady state at *c*_A_ = 0.2 for various bond flexibility of A species (bond flexibility of B is always *p*_flex_ = 1.0). Only A species is shown (red spheres) for visual clarity. (c) Corresponding snapshots are shown by swapping the A and B compositions (*c*_A_ = 0.8). Only B is shown by obscuring A monomers for visual clarity. Snapshots of full systems with both species are shown in Figure S1.

The system composition is determined by identifying the fraction of A species *c*_A_ = *N*_A_/*N*_tot_ from the total number of spheres *N*_tot_, and *c*_A_ varies from 0.1 to 0.5. Periodic boundary conditions are implemented in the BCD model. A and B particles undergo intraspecies interaction through short-range square well potential with an interaction range ϵ = 0.1. The interspecies interactions are through hard-core repulsion. The schematic of the model system is shown in Figure 1(a). When the center-of-mass distance between A–A and B–B is within the distance of 1 + ϵ, particles form irreversible bonds. The collection of all bonded particles is defined as clusters of either the A or B species. The movement of both *A* and *B* monomers is rejected if it results in either overlapping with another monomer or separating the bound monomers beyond the interaction range.

As monomers diffuse, the center-of-mass of the cluster they comprise would also be displaced in a random direction mimicking Rouse dynamics.^26^ The center-of-mass of each cluster, except for clusters of size one, undergoes an additional displacement in the same direction as that of the Rouse dynamics with a diffusion coefficient inversely proportional to the cluster diameter, mimicking Zimm dynamics.^27^ Hydrodynamic interactions are not included in the present study. It has been reported elsewhere that BCD’s structure, kinetics, and dynamics show good agreement with event-driven molecular dynamics for monomeric systems.^28^ The key advantage of the BCD method is that it allows us to perform simulations for a very large system size of ≈10^5^ particles within reasonable computational time. The details of the BCD model are provided in the Supporting Information.

## Results

The snapshots of the binary system are shown at a constant composition of *c*_A_ = 0.2 (*c*_B_ = 0.8) in Figure 1(b) and *c*_B_ = 0.2 (*c*_A_ = 0.8) in Figure 1(c) for ϕ_tot_ = 0.3 at a range of *p*_flex_. The snapshots are taken at *t*/*t*_0_ = 1.496 × 10^3^ when the value of mass-averaged aggregation number (*m*_w_) for A in Figure 1(b) and B in Figure 1(c) stabilizes to a stationary value. At *p*_flex_ = 0.0, when the bonds are fully rigid, small aggregated clusters are distributed throughout the simulation box, and A species do not form percolated clusters. This system remains in the flocculation regime, characterized by a fractal dimension of 2.^24^ However, with increasing *p*_flex_, the cluster densifies and more free space is visible in the system. To visualize this effect, the A species is shown in the absence of B at the top row in the red box (the snapshots of these systems with both species are shown in Figure S1). For *p*_flex_ > 0, the local structure remains the same as flexibility only enhances the kinetics of aggregation, and the clusters formed are nearly the same. At *c*_A_ = 0.8 and *p*_flex_ = 0 (Figure 1(c)), we observe that the clusters formed by the B species are apart from each other, and locally, they have more open structures compared to systems with higher *p*_flex_ as seen in the snapshots of the green box. While the structures look similar, the cluster kinetics are different, as shown in Figure S2.

### Appearance of Bigel

**Figure 2:**
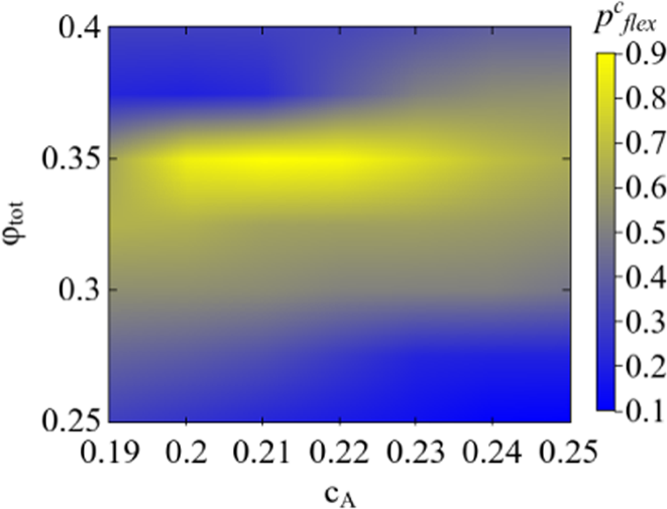
State diagram visualized with a heat map between ϕ_tot_, *c*_A_, and *p*_flex_^c^. The color gradient represents the flexibility parameter *p*_flex_. The presence of bright blue colors at the bottom-right and top-left corners demonstrates that at moderate volume fraction and at very high volume fraction, A clusters percolate with stiffer bonds. However, the emergence of yellow colors indicates that A clusters percolate with a higher flexibility of the bonds. Here, *c*_A_ represents the critical concentrations at which the appearance of bigels is observed in binary systems.

In a binary colloidal system, a gel is identified as a cluster that percolates or, in other words, spans from one end of the box to the opposite end. In this study, the systems forming the one-component gel or bigel are identified by performing the simulation for 10 independent initial configurations by varying *p*_flex_ at a range of *c*_A_ for various volume fractions in a box size of *L* = 50. If at least half of the simulated systems result in the percolating network of B species and finite (nonpercolating) clusters of A monomers, we consider such a system as the one-component gel. When both A and B species form percolated clusters, the system is considered to be a bigel. In the present work, we have determined the range of composition *c*_A_ and bond flexibility *p*_flex_ at various volume fractions where the system will form either a one-component gel or bigel. The range of *c*_A_ determined at various volume fractions is defined as the critical concentration at which the bigel starts appearing in the system from a particular *p*_flex_. In Figure 2, a heat map of the state diagram is presented where the bigel formation is shown for total volume fraction (ϕ_tot_), the composition of *A* species *c*_A_, and the critical bond flexibility parameter *p*_flex_^c^, which is represented by the color gradient. For instance, for ϕ_tot_ = 0.40, *c*_A_ = 0.19 ± 0.01 at *p*_flex_ < 0.3 system resulted in a one-component gel but with the same composition of *c*_A_ and *p*_flex_ ≥ 0.3 bigel appears in the system. The combination of *c*_A_, ϕ_tot_, and *p*_flex_ was further confirmed by performing simulations at *p*_flex_ > *p*_flex_^c^ for same *c*_A_ and ϕ_tot_, where we always observed bigel. It is important to note that the effect of bond rigidity on aggregation kinetics at ϕ_tot_ < 0.25 was negligible, and the (*c*_A_)_c_ is found to be consistent with a previous study where bonds are fully flexible for both species.^24^ In the case of fully flexible bonds, the transition from the one-component system to a bigel system is dictated only by the critical concentration. In the present work, the transition to bigel happens at different *c*_A_ values as ϕ_tot_ is varied according to the bond flexibility. However, the bond flexibility increases the critical concentrations for ϕ_tot_ > 0.25 and ϕ_tot_ < 0.25, where (*c*_A_)_c_–*c*_A_/(*c*_A_)_c_ remains the critical parameter for irreversible aggregation of a binary system consistent with percolation theory.^24^ Bond flexibility does not play a major role in the bigel formation in low-volume fraction binary systems. In this case, the monomers are distributed far apart and take more time to diffuse around and form bonds, and by this time, the monomers have enough time to locally rearrange, whereas rigid bond clusters keep spreading their branches to form the percolating clusters. The critical concentrations ((*c*_A_)_c_) increase with decreasing volume fractions, meaning that for the appearance of bigel, more A monomers are required, which leads to a less dense percolated network of B species. This in turn leads to more accessible volume to rearrange for the flexible B clusters around A clusters and therefore the critical *c*_A_ is unaffected by bond rigidity. Thus, the critical concentrations remain unchanged in moderate volume fractions at various *p*_flex_ and explain the limited range of ϕ_tot_ and *c*_A_ in Figure 2.

It is seen from Figure 2 that with increasing volume fraction ϕ_tot_, *p*_flex_^c^ increased for the bigel to be formed. For a very low ϕ_tot_, the A species form clusters easily as the available free volume is ample. Also, it is shown elsewhere that the clusters formed by rigid bonds occupy more space compared to clusters with flexible bonds.^28^ As ϕ_tot_ is increased, the A clusters get stuck inside the cages formed by the B species cluster. The cages of the percolating cluster of the B species are dynamic, which means the cages open and close due to the dangling ends of the percolating clusters. Thus, the rigid bond cluster, which occupies more space, will get stuck inside the cages when the cluster size of A is bigger than B clusters. To escape the cage confinement, the A cluster needs to reduce its spread, which is possible by increasing the bond flexibility that locally densifies the clusters. Therefore, as ϕ_tot_ increases, the bigel is formed for the increased flexibility of the bonds for the same *c*_A_. For very high ϕ_tot_, the average size of the cage decreases due to the increased number of particles. The A species then aggregate out of the cages, and even less flexibility leads to the bigel system. This effect is apparent at volume fraction 0.40, where *p*_flex_^c^ decreases, as is visible by the appearance of blue color in the top-left corner of the plot in Figure 2. Moreover, it is essential to note that the critical concentration parameter (*c*_A_)_c_ has increased for a binary system with *p*_flex_ < 1.0^24^ as shown in Table S1. Since the flexibility is varied only in A species, and B species are aggregating with *p*_flex_ = 1.0, we also investigated the aggregation mechanism of B species at *c*_A_ ≥ 0.5, i.e., *c*_B_ ≤ 0.5. It is consistent with the earlier findings when A and B species aggregated with full bond flexibility.^24^

### Kinetics under the Influence of Bond Rigidity

**Figure 3:**
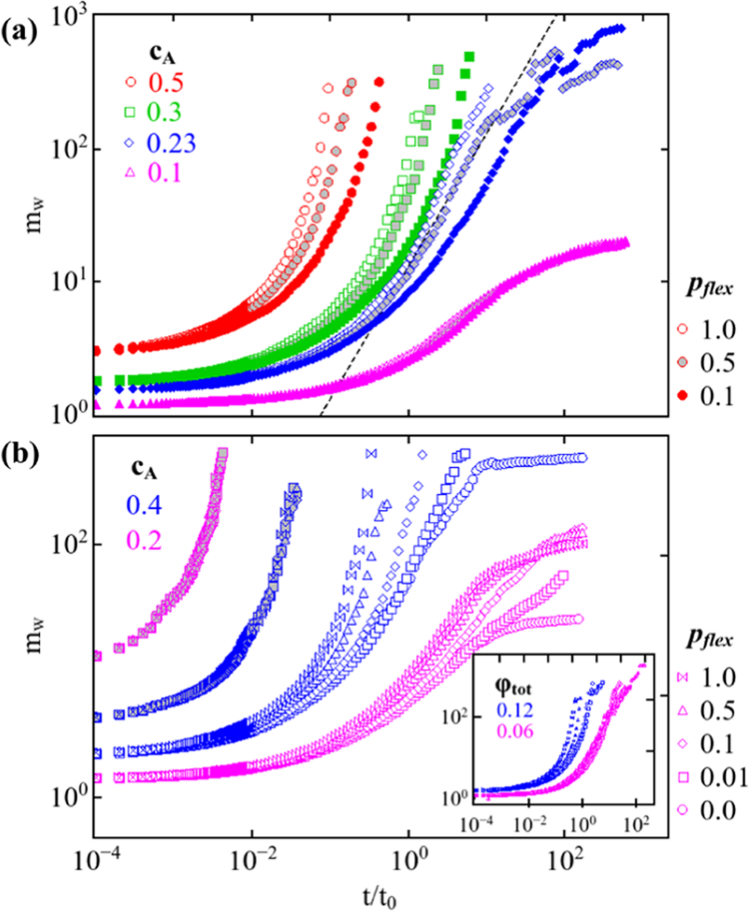
(a) Mass-average aggregation number (*m*_w_) is plotted as a function of reduced time for ϕ_tot_ = 0.3 at various *c*_A_ for *p*_flex_ = 0.1 (open symbols), 0.5 (gray filled symbols), and 1.0 (filled symbols). The dashed line indicates the slope of unity in accordance with the Smoluchowski rate equation. (b) *m*_w_ is shown for *c*_A_ = 0.4 and 0.2 at a range of *p*_flex_ for both A (open symbols) and B (gray filled symbols) species. In the inset, *m*_w_ is visualized for the monomeric system, where all of the particles are of the same species.

To understand the kinetics of the controlled aggregation mechanism in the binary colloidal system, we calculated the mass-average aggregation number given as eq 2 and plotted in Figure 3
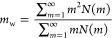
2

Figure 3(a) presents the evolution of *m*_w_ for only *A* species as a function of reduced time for ϕ_tot_ = 0.3 at a range of *c*_A_ and *p*_flex_ values. For the initial time (*t*/*t*_0_ < 10^–2^), the aggregation kinetics for a particular *c*_A_ value at different flexibility values collapse on top of each other, and cluster growth is independent of bond flexibility. At a later time, the A species aggregation is influenced by the presence of B species, and the curve with *p*_flex_ = 1.0 forms a percolating cluster faster, as *m*_W_ diverges at an earlier time for (*c*_A_)_c_ ≤ 0.23, as shown in Figure 3(a). It is a known fact that in DLCA aggregation, the *m*_w_ will grow linearly with time for a small volume fraction.^29^ In Figure 3(a), we observe that close to the critical concentration and at moderate flexibility, a slope of unity is observed (shown by a dashed line). Below the critical concentration of *A* species, i.e., (*c*_A_)_c_ ≤ 0.23 *m*_w_ falls below a slope of 1, indicating finite cluster formation; above the critical value, the slope of *m*_w_ moves above unity, indicating the formation of an interpenetrated network that spans the system. This is consistent with a single component system where at ϕ_tot_ > 0.1, the system always forms percolated clusters before the monomers are able to diffuse their own diameters, resulting in the gel time *t*_g_ < 1.^30^ For low A composition, *c*_A_ = 0.1, it is notable that for all *p*_flex_, *m*_w_ approaches the same stationary value because the size of the percolated B clusters is the same. Interestingly, even the kinetics of the A species are nearly independent of flexibility because the time taken for A particles to diffuse to form bonds is greater than the bond relaxation time.

Furthermore, to explore the effect on the aggregation of B by varying the bond flexibility in A species, we analyzed the growth kinetics of both A and B clusters. For the analysis of growth kinetics, systems with two different compositions (*c*_A_ = 0.2 and 0.4) are chosen. The system with *c*_A_ = 0.2 is below the critical concentration ((*c*_A_)_c_ = 0.23) and the system composition with *c*_A_ = 0.4 represents the system above the critical concentration of bigel formation. The evolution of the cluster growth is shown by plotting the mass-averaged aggregation number as a function of reduced time for a range of *p*_flex_ values shown in Figure 3(b). Varying bond flexibility in A does not have a significant effect on the aggregation of B as *m*_w_ for B species (shown by gray filled symbols) falls on top of each other for 1.0 ≥ *p*_flex_ ≥ 0.0 at both *c*_A_ values. However, aggregation of A species strongly depends on bond flexibility, and it is observed that even above a critical concentration of A species (i.e., *c*_A_ = 0.4), *m*_w_ approaches a stationary value at *p*_flex_ = 0.0. Moreover, the critical *c*_A_ for zero flexibility at ϕ_tot_ = 0.3 is observed to be significantly higher, i.e., 0.8 > (*c*_A_)_c_ > 0.7. This demonstrates that when the bonds are completely rigid for *A* species, the binary system results in a one-component gel. It is remarkable that albeit 1% of flexibility in bonds is significant for the system to result in a bigel, as observed at *c*_A_ = 0.4, *p*_flex_ = 0.01 in Figure 3(b). The bond flexibility also affects the growth kinetics of finite A clusters, which aggregate in the cages of the percolated B cluster. Also, below the critical concentration, with decreasing *p*_flex_, *m*_w_ starts approaching the stationary value and remains almost constant.

In the inset of Figure 3(b), the evolution of *m*_w_ for the monomeric systems at ϕ_tot_ = 0.12 and 0.06 is shown. These monomeric systems have the same volume fraction as that for only A species at *c*_A_ = 0.4 and 0.2 in a ϕ_tot_ = 0.3 binary system. In a monomeric system at a 0.12 volume fraction, the influence of bond flexibility is apparent in terms of delay in the gelation. However, a crossover is observed at 0.06 volume fraction as *p*_flex_ decreases. At a small volume fraction, the crossover of *m*_w_ with reduced bond flexibility is consistent with the previous finding.^29^ In the binary case, the crossover of *m*_w_ is absent for the concentration *c*_A_ = 0.2; instead, cluster growth stagnates, resulting in stationary values of *m*_w_, thus, forming only finite clusters.

### Self-Similarity and Lattice Animals

The self-similarity of clusters is determined by the fractal dimension *d*_f_ by the power law dependence of mass aggregation number *m* with the radius of gyration *R*_g_ of the cluster, given as
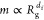
3

**Figure 4:**
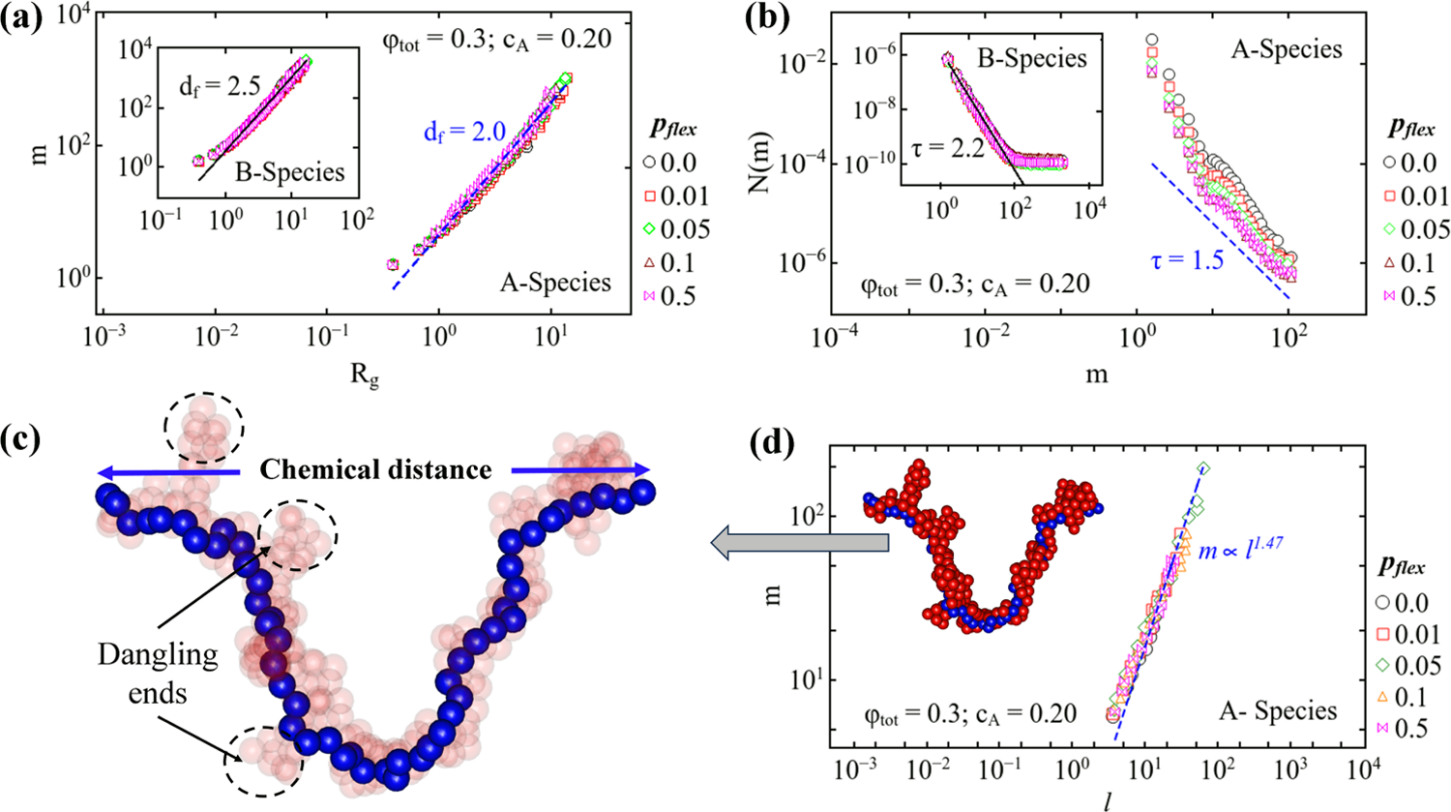
(a) Aggregation number *m* is plotted as a function of radius of gyration *R*_g_ for A and B (in inset) species at a range of *p*_flex_. The dashed and solid lines represent the slope, i.e., fractal dimension *d*_f_ = 2.0 for A and 2.5 for B, respectively. (b) Size distribution of clusters *N*(*m*) is plotted as a function of the aggregation number *m* corresponding to flexibility and system composition shown in panel (a). The dashed line has a slope of 1.5, i.e., *N*(*m*) ∝ *m*^–τ=1.5^ for *A* species. For *B*, the exponent τ = 2.2 is shown by the solid line in the inset. (c) Snapshot of one cluster, which is isolated from the system to compute chemical distance. The chemical distance is the length of the path between two monomers at the ends. For visual clarity, the whole cluster is shown in translucent red with the path (linear chain) in blue, and the solid color representation of the same cluster is shown in the inset of panel (d). The dashed line in panel (d) has the slope of 1.47, confirming the presence of lattice animals at all *p*_flex_ in a system composition of ϕ_tot_ = 0.3, *c*_A_ = 0.20.

For the system of one-component diffusion-limited cluster aggregation (DLCA), it is well-known that the clusters are self-similar to fractal dimensions 1.8 (flocculation regime) and 2.5 (percolation regime).^31^ In Figure 4, the aggregation number *m* is plotted as a function of the radius of gyration *R*_g_ for A and B (inset) species, respectively. The fractal dimension is determined by the power law dependence for both species at a range of *p*_flex_ values. For *A* species, the exponent *d*_f_ is determined to be 2.0 shown by the dashed line in Figure 4(a). Generally, *d*_f_ = 2.0 is reported in the reversible aggregation of colloids as well as during the formation of branched polymers (or lattice animals).^32,33^ Also, it has long been accepted that in dilute limit, the branched polymers and lattice animals are in the same universality class.^34^ Generally, the lattice animal is defined as clusters of connected sites on a regular lattice. In this study, off-lattice simulations of binary colloids are performed; however, it has been previously reported that the fractal dimension of aggregating clusters obtained on- and off-lattice for irreversible DLCA is the same as random percolation.^35,36^ Therefore, in this context, the term “lattice animal” is adapted for the off-lattice BCD model. According to the percolation theory, the lattice animal appears far before the percolation threshold. Because of the selective intraspecies attractive interactions, every collision leads to bond formations, and clusters thus formed densify before experiencing collision, and interspecies collisions do not lead to bond formation. Therefore, the structure of A is influenced by the hindrance effect caused by the B species. The hindrance effect on cluster structures of A and B is best realized in the case of 50–50 composition of both species at a low-to-moderate volume fraction.^24^ The increased fractal dimension indicates that irreversible aggregation leads to self-similar clusters for which all configurations for a given aggregation number are equiprobable for both species before the percolation threshold. Additionally, a fractal dimension of 2.0 is expected when each configuration is equally probable like in the case of reversible aggregation where clusters rearrange swiftly compared to their diffusion rate.^37^ Since the effect of bond rigidity is negligible, below ϕ_tot_ < 0.25, the fractal dimensions are consistent with our previous findings.^24^ In the present work, bond flexibility is tuned only for A particles and kinetics are altered significantly below the critical concentration; for this reason, we chose the system at ϕ_tot_ = 0.3, *c*_A_ = 0.20 for fractal dimension analysis. The A clusters at all *p*_flex_ are self-similar to *d*_f_ = 2.0 and clusters remain lattice animals (in flocculation limit), which is also indicated by the approaching stationary *m*_w_ at *c*_A_ = 0.2 in Figure 3(b). At *c*_A_ = 0.2, the clusters of B species are self-similar to *d*_f_ = 2.5 shown by the solid line in the inset of Figure 4(a). For B particles, the analysis is done just before the percolation threshold when clusters are already in the percolation regime and are no longer influenced by the bond rigidity in A particles. In addition, for all flexibility values, the clusters in the system compositions above the critical concentration are found to be self-similar to the fractal dimension of 2.0 in the flocculation limit and transition through to 2.5 in the percolation limit; this is consistent with a previous study where bonds are fully flexible for both species.^24^

Further, we also analyzed the cluster size distribution at *p*_flex_ values for both A and B clusters for different system compositions. The cluster size distribution *N*(*m*) is described by the power law decay with an exponential cutoff.^30^ We know that *N*(*m*) ∝ *m*^–τ^ and the exponent τ are determined numerically for 3D percolation. Traditionally, in the flocculation limit, the diffusion-limited cluster aggregation is characterized by τ = 0 with *d*_f_ = 1.8, while for lattice animals, τ = 1.5 with *d*_f_ = 2.0 is observed.^36^ In Figure 4(b), the cluster size distribution is presented when the system is in the early stages of aggregation and away from the percolation threshold. The dashed line represents the slope of 1.5, i.e., *N*(*m*) ∝ *m*^–τ=1.5^ as predicted by the percolation theory for lattice animals. The exponent τ = 1.5 confirms that A species have a fractal dimension of 2.0. In the inset of Figure 4(b), the cluster size distribution is shown for the B species that forms the percolating clusters and is confirmed by τ = 2.2 with a slope given by a solid line. Furthermore, lattice animals are observed for both A and B clusters in a system composition above the critical concentration in low-to-moderate volume fractions, where the crossover from lattice animals to percolation is marked by the transition of the fractal dimension 2.0–2.5.

Conventionally, reaction limited cluster aggregation (RLCA) is distinguished from DLCA with *m* ∝ *R*_g_^*d*_f_ = 2.0^ and *N*(*m*) ∝ *m*^–τ=1.0^. To differentiate the structures observed in RLCA systems and lattice animals in this study, we further analyze the chemical distance (similar to the end-to-end distance in polymer models^38^) in A clusters at all flexibility values. The chemical distance *l* is the length of the shortest path between two monomers of a cluster. To determine *l* in A species, clusters that have fractal dimension 2 (away from the percolation threshold) are isolated from the binary system, and one such snapshot is shown in Figure 4(d). In the isolated cluster, one monomer is identified to calculate a distance from the other monomer that is at the farthest point in the cluster. After finding two monomers, the cluster is reconstructed by removing one of the monomers to confirm if the two identified monomers belong to the same cluster. If both the monomers are of the same cluster, it means the monomer that is removed is not part of the dangling ends, and then the dangling ends (encircled in Figure 4(c)) are removed from the cluster. We repeat the process for all monomers, which results in a linear chain of monomers (shown in blue color in Figure 4(c)), which is the chemical distance of that cluster. In the study of irreversible “nondeletion reaction limited cluster–cluster aggregation (NDRLCA)”, it is shown that the backbone dimension (*d*_b_) of the RLCA cluster is ≈1 and for lattice animals, *d*_b_ = 1.36. In Figure 4(d), the aggregation number *m* is plotted as a function of *l* and the scaling exponent is given as *d*_f_/*d*_b_.^34^ For lattice animals, *m* ∝ *l*^*d*_f_/*d*_b_ = 1.47^ is shown by the dashed line. For all *p*_flex_, the data collapse on top of each other, indicating that lattice animals are present in all systems irrespective of the bond rigidity. Additionally, if the self-similarity of the binary cluster with *d*_f_ = 2.0 is to be associated with RLCA, then *d*_f_/*d*_b_ is expected to be 2.0 instead of 1.47. Therefore, our analysis confirms the presence of lattice animals at all bond flexibility values.

### Nearest-Neighbors in Rigid Bond Clusters

**Figure 5:**
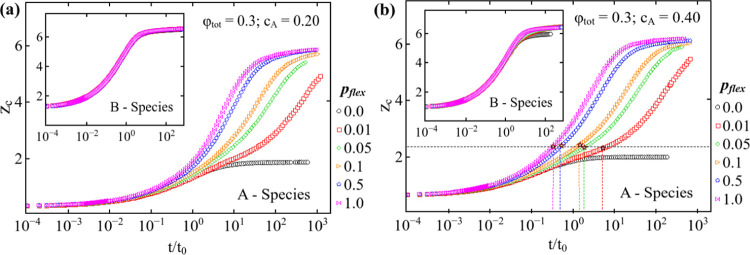
Average number of bonded neighbors *Z*_c_ is plotted as a function of reduced time for ϕ_tot_ = 0.30 at (a) *c*_A_ = 0.40 and (b) 0.20 for A species at various *p*_flex_ values. Insets show the average number of bonded neighbors for *B* species for the respective *c*_A_ and *p*_flex_. In panel (b), the vertical dashed lines indicate the gel time as the bond flexibility varies. The horizontal dashed line indicates that the average value of *Z*_c_ is approximately the same at all *p*_flex_, and star symbols represent the average *Z*_c_ at the gel time for every *p*_flex_ separately.

Figure 5 presents the average number of bonded neighbors, *Z*_c_, for both species. It is established in an earlier study that in the irreversible colloidal system with *p*_flex_ = 1, the aggregation leads to an increase in the number of bonds per monomer^29^ compared to the *p*_flex_ = 0 system. The average number of bonded neighbors are plotted as a function of reduced time for A species at *c*_A_ = 0.2 (Figure 5(a)) and 0.4 (Figure 5(b)) at a range of flexibility values. *Z*_c_ values for B species are shown in the insets of Figure 5. At the initial time, *Z*_c_ is the same at all *p*_flex_ as the same number of *A* monomers distributed in the binary system. As the cluster grows, the flexibility of bonds comes into play, and *Z*_c_ increases sharply before approaching a stationary value at approximately 6 for the A species for the *p*_flex_ = 1 system. At reduced flexibility or increased rigidity, *Z*_c_ increases steadily before plateauing at approximately the same value except for *p*_flex_ = 0.0. In the case of systems with 0.0 < *p*_flex_ < 0.1, the dangling ends of A clusters grow slowly and only due to rearrangement of B around them because portions of A get stuck inside the percolated B network. Therefore, averaged *Z*_c_ at flexibility values between 0.0 and 0.1 show the trend of approaching a plateau but at a longer time scale. However, within reasonable computational time, plateauing of *Z*_c_ for rigid bond clusters below the critical concentration may not be realizable for *L* = 50. At *p*_flex_ = 0.0, i.e., when the bonds are completely rigid, the number of average neighbors is close to 2. This trend is similar at both *c*_A_ values that are below (*c*_A_ = 0.2 in Figure 5(a)) and above (*c*_A_ = 0.4 in Figure 5(b)) the critical concentration of A species for the volume fractions we analyzed. When the center-of-mass of two spheres is within 1 + ϵ, a bond is formed, and rearrangement within the bonds is not allowed for rigid bonds. Thus, we only observe monomers with less than 2 neighbors for low-volume fractions. When the flexibility is tuned, the particle rearranges within the bonds and maximizes the number of bonds. There is only a delay in the system’s kinetics for small flexibility compared to *p*_flex_ = 1. Unlike fully rigid cases, all systems approach the same steady-state value of approximately 6 for A. Furthermore, it is interesting to note that at *c*_A_ = 0.2, i.e., *c*_B_ = 0.8, the average number of neighbors in B clusters are the same for various *p*_flex_ in A species. However, at *c*_A_ = 0.4, i.e, *c*_B_ = 0.6, *Z*_c_ plateaus at slightly different values. These values are observed to be higher than 6 (*Z*_c_ is shown for B in the insets) except for *p*_flex_ = 0. This indicates that tuning the flexibility and concentration of the A species potentially enables control over the structure of the B species.

## Discussion and Conclusions

We demonstrate that flexibility can play a pivotal role in altering the aggregation kinetics of colloidal mixtures. In a binary colloidal mixture, as bond flexibility decreases, rigid bonds delay the gel formation. This finding complements the recent work of Howard et al.^11^ on the colloid–linker mixture, where the gelation was suppressed by allowing the linkers to form the ”loop” on the same colloid particle. In contrast to the critical concentration being the only parameter for colloidal aggregators to form aggregates, our study highlights the underlying interplay among critical concentration, flexibility, and packing volume fraction. The hypothesis of tuning the aggregation mechanism is coherent with the experiment in which controlled aggregation was achieved by adding the gold nanoparticles (AuNPs) in a mixture of the end-functional stabilizing polymer and a hydrophobic aggregating agent. Both the agents bind to the gold nanoparticles, and by varying the ratio of the agents, the degree of aggregation was tuned.^39^ The aggregation that could be controlled in experimental systems was also realized by a carefully crafted system of a crystalline monolayer of latex particles with combinations of the electrolyte and surfactant. The electrolyte and surfactant enable control over the fractal dimension and kinetics of aggregation.^40^ It is important to note that in this study, the fractal dimension was analyzed just before the percolation threshold and at stationary *m*_w_, it is found to be 2.0, which is consistent with the binary colloids and signifies the presence of lattice animals in the binary DLCA system.^24^ Moreover, it is necessary to note that the experimental systems and model presented in this study cannot be directly compared due to the binary mixture’s specific characteristics, wherein both species are of equal size. The discussion here is indicative of the potential for tuning the control in the aggregation mechanism.

Furthermore, we observed that with varying flexibility, the critical concentration increases by at least 1–2% for all packing volume fractions. However, the effect of rigidity is observed not only in terms of increased critical concentration but also in two scenarios: (i) above the critical concentration and (ii) below the critical concentration. In the first case, the rigidity in bonds affects the aggregation steps (oligomers, aggregates, and gel) by delaying dynamic arrest in the A species. In the second scenario, the bond rigidity influences the cluster size as observed with various stationary *m*_w_ for the same *c*_A_. It is important to note that this effect is limited for *c*_A_ only up to 3–5% below (*c*_A_)_c_, and the effect disappears at very small concentrations. The other key highlight of the effect of bond rigidity in scenario one is that with increasing rigidity in bonds, the gel time increases but the number of averaged neighbors is roughly the same. However, in the second scenario, the trend of averaged neighbors is similar to scenario one, except that gelation does not occur.

In summary, the presented findings open routes for further exploration of the role of the binding mechanism in colloidal aggregation, with foreseeable implications in fields such as early drug discovery research. While colloidal aggregates of small molecules have traditionally been regarded as a nuisance artifact in drug screening, recent efforts to leverage their distinctive properties are shifting this perception. The use of excipients such as polymers, proteins, and other small-molecule aggregators are studied to stabilize the colloidal aggregates and their potential use as intentional drug formulations.^41,42^ We anticipate that the findings of this study will add to discussion in the same direction.

Moreover, it remains to be determined how bond rigidity influences both the species’ dynamics and diffusive behavior. By varying the attraction strength, one can also untangle the roles of the interaction range with rigid and flexible bonds in binary systems. The athermal irreversible system leads to the gel formation for all *p*_flex_ above critical concentrations; hence, underpinning the role of *p*_flex_ at a finite temperature as well as consideration of the size ratio is essential for practical applications.

## Data Availability

The data that support the findings of this study are available in the Supporting Information.

## Supporting Information Available

Detailed description of Brownian Cluster Dynamics for binary colloids; snapshots of binary systems with both species present along with kinetics of the systems in terms of nearest-neighbors for rigid clusters; and tabulation of determined critical concentrations at a range of volume fractions (PDF)
