# Supplementary material for: Rigidity-Induced Controlled Aggregation of Binary Colloids
Source: ACS Omega. 2023 Sep 27;8(40):37225–32. doi: 10.1021/acsomega.3c04909 (PMC10568703; doi:10.1021/acsomega.3c04909)
Supplement: Supplementary file 1 — ao3c04909_si_001.pdf [file ao3c04909_si_001.pdf]

Rigidity induced controlled aggregation of binary colloids

Zakiya Shireen,[*,†] Tine Curk,[‡] Christian Brandl,[†] and Sujin B Babu[*,¶]

†*Department of Mechanical Engineering, Faculty of Engineering and Information Technology, University of Melbourne, Victoria, Australia*

‡*Department of Materials Science and Engineering, Whiting School of Engineering, Johns Hopkins University, Maryland, USA*

¶*Out of Equilibrium Group, Department of Physics, Indian Institute of Technology Delhi, New Delhi, India*

E-mail: zakiya.shireen@unimelb.edu.au; sujin@physics.iitd.ac.in

Model and Method

Brownian cluster dynamics (BCD) is based on the Metropolis Monte Carlo method and inspired by the work of Meakin et al.[1,2] and Kolb et al.,.[3] It starts with the initial configuration of N_{tot} randomly distributed hard-spheres in a 3-dimensional box of size L. The total volume fraction of the system is given as $\phi_{tot} = \frac{\pi}{6} N_{tot}/L^3$. The composition of the binary system is defined by identifying a fraction of A monomers with the concentration $c_A = N_A/N_{tot}$, where N_A is the number of monomer of A species. Then the concentration of B monomers is given by $c_B = 1 - c_A$. For both the species, the monomers are considered to be bonded when they are within each other's interaction range, i.e., when the center-to-center distance between the same species of monomers is $\leq (1 + \epsilon)$, where ϵ is the interaction range. Thus, clusters are collections of bound monomers, and lone monomers are clusters of size 1, resulting in N_c

number of clusters in the system. For the cluster construction, monomers are randomly chosen N_{tot} times, and each monomer is displaced randomly with a step size $s = 0.01$, chosen to be at least 10 times smaller than the ϵ to mimic the Brownian motion .[4]The movement step is rejected if it overlaps with another monomer or leads to a separation of bound monomers beyond interaction range. The center-of-mass displacement of the clusters is calculated, and the clusters move cooperatively in the same direction so that the total displacement is inversely proportional to their radius. Upon the cluster construction and the movement step, the time is incremented as $t = ns^2$ where n is the number of simulation steps. Thus, $t = 1$ is the time taken by a monomer to travel its own diameter, where the bare diffusion coefficient of monomer is defined as $D_0 = 1/6$.[4] It is to underline that BCD is equivalent to Brownian Dynamics if the cooperative cluster displacement step is omitted, given that step size s should be sufficiently small.

Binary system

In Figure S1, the snapshots of the modeled system is shown for a composition of 20% and 80% of A and B monomers. The red and green represents A and B monomers, respectively. A system with constant composition ($c_A = 0.2$; $c_B = 0.8$) is simulated for various values of flexibility parameter p_{flex} and the snapshots are shown in Figure S1(a). In Figure S1(b) a binary system is simulated by swapping the composition of A and B, i.e., only 20% of monomers are of B species and remaining are A ($c_B = 0.2$; $c_A = 0.8$). In both Figure S1(a) and (b), the flexibility is tuned only in A species, and bonds are fully flexible in B. For both compositions, the snapshots are taken at $t/t_0 = 1.496 \times 10^3$ when the cluster growth stagnates. It is important to note that $c_A = c_B = 0.2$ is below the critical concentration of percolation in binary system ;[5] therefore, the value of m_w stagnates for A in Figure S1(a) and B in Figure S1(b).

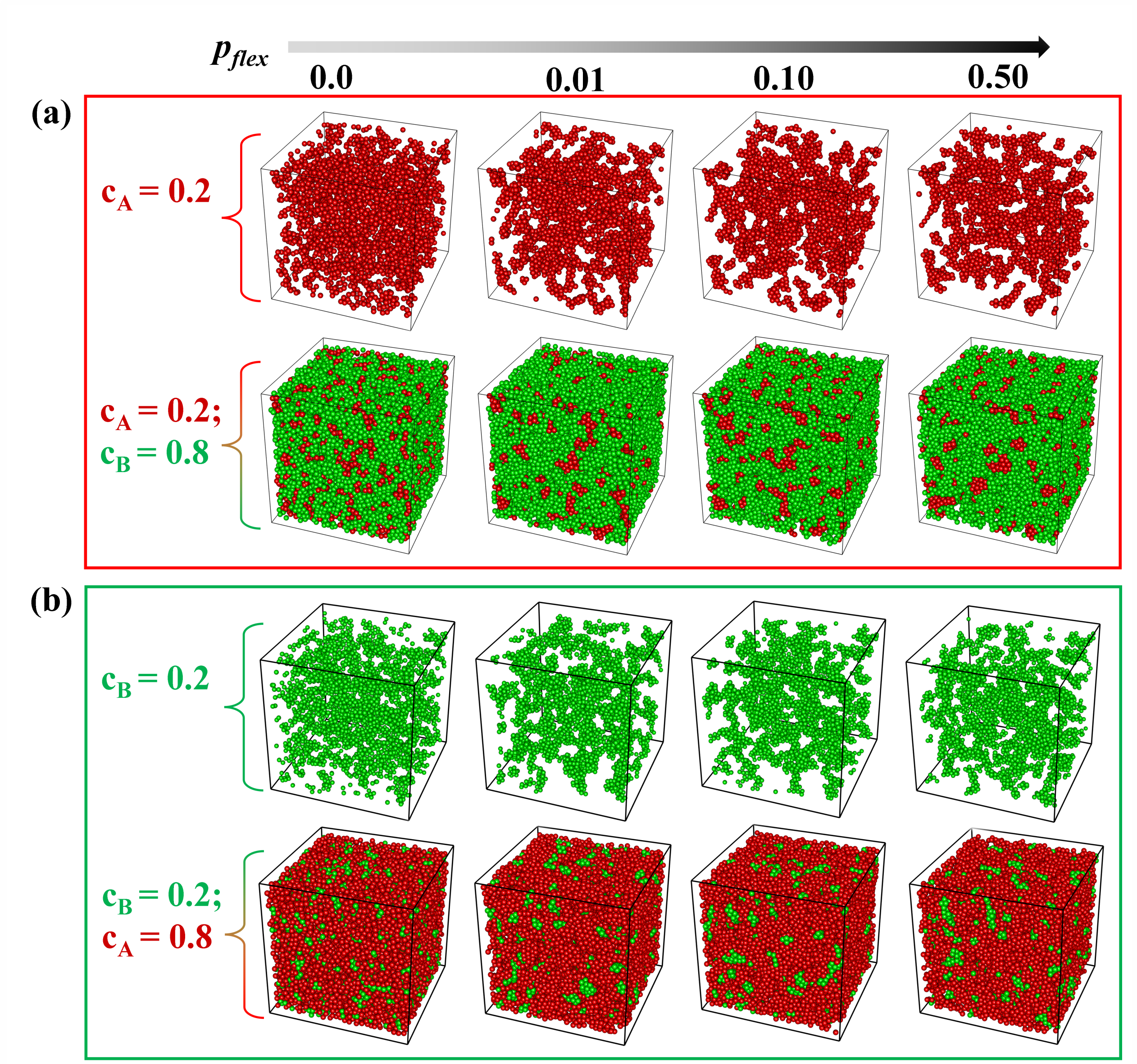

Figure S1: (a) Snapshots of the binary system are shown for varying bond flexibility in A species, and bonds are fully flexible for B. In the top row, A species is shown in the absence of B, and bottom row, A, and B both are shown. (b) The snapshots in the green box are shown by swapping the A and B compositions, but the bond flexibility is tuned only in A. In the top row, only B species are shown, and in the bottom row, the snapshot of the full system is shown with A and B both.

Kinetics

In Figure S2 the averaged number of bonded neighbor is analyzed for $c_A = c_B = 0.2$ corresponding to system presented in Figure 1 (b) and (c) at a range of p_{flex}. These concentrations are simulated in total volume fraction of $\phi_{tot} = 0.3$ with flexibility tuned only in A. The difference in the evolution of Z_c for is prominent for the fully rigid bonds (Figure S2(a)). As we increase the flexibility in A the average number of bonded neighbors plateau roughly at the same value as that of B species.

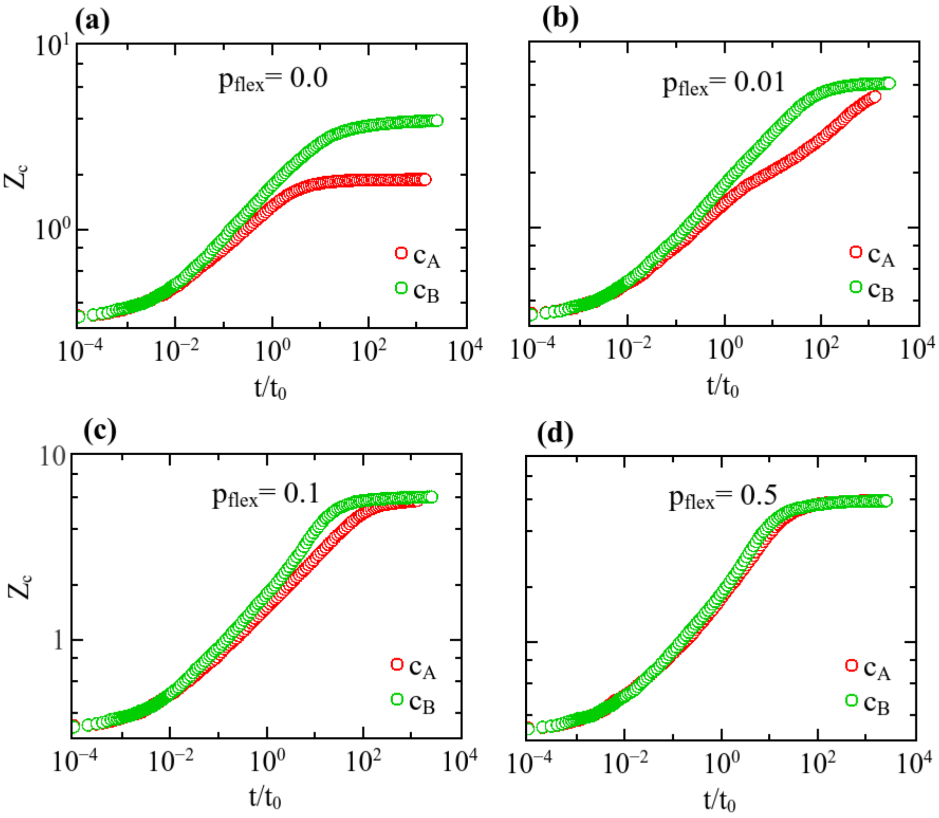

Figure S2: The nearest neighbor of both A and B is shown to highlight the difference in cluster growth with time at range of $p_{flex} = 0.0$(a), 0.01(b), 0.1(c) and 0.5(d), respectively. The Z_c shown here corresponds to the Figure 1 (b) and (c) in the main text.

In the state diagram in the main text, the range of ϕ_{tot}, c_A and p_{flex} represent binary systems in which bigel start appearing at a particular $p_f lex$ for a given ϕ_{tot}, c_A. For example, at $\phi_{tot} = 0.40$, $c_A = 0.19 \pm 0.01$ at $p_{flex} < 0.3$ system resulted into a 1 component gel but with the same composition of c_A and $p_{flex} \geq 0.3$ bigel appears in the system. The combination of

Table S1: Determined critical concentrations of A species $((c_A)_c)$ at $0.0 \geq p_{flex} \leq 1.0$.

	at $p_{flex} < 1.0$ (present work)		at $p_{flex} = 1.0$ (literature)[5]	
ϕ_{tot}	$(c_A)_c$	N_A	$(c_A)_c$	N_A
0.25	0.25	14920	0.24	14323
0.30	0.23	16472	0.22	15756
0.35	0.20	16711	0.19	15875
0.40	0.18	17188	0.17	16233

c_A, ϕ_{tot} and p_{flex} was further confirmed by performing 10 independent simulations at $p_{flex} > p^c_{flex}$ for same c_A and ϕ_{tot}. Out of all simulations, in more than 50% systems, we observed bigels. With reduced bond flexibility, it is observed that the critical concentration parameter $(c_A)_c$ for a bigel to appear increases. Which is higher than the previously reported c_A values .[5] For comparison the critical c_A values $((c_A)_c)$ are tabulated in Table S1. Though the difference in c_A values is about 1% however, the number of monomers varies significantly. In Table S1, the corresponding number of A monomers (N_A) are also tabulated along with critical c_A. It is essential to note that the difference of one monomer determines the percolated or non-percolated cluster in finite box size.

References

(1) Meakin, P. Formation of fractal clusters and networks by irreversible diffusion-limited aggregation. *Physical Review Letters* **1983**, *51*, 1119.

(2) Meakin, P. Diffusion-controlled cluster formation in 2—6-dimensional space. *Physical Review A* **1983**, *27*, 1495.

(3) Kolb, M.; Botet, R.; Jullien, R. Scaling of kinetically growing clusters. *Physical Review Letters* **1983**, *51*, 1123.

(4) Babu, S.; Gimel, J.-C.; Nicolai, T.; De Michele, C. The influence of bond rigidity and

cluster diffusion on the self-diffusion of hard spheres with square well interaction. *The Journal of Chemical Physics* **2008**, *128*, 204504.

(5) Shireen, Z.; Babu, S. B. Lattice animals in diffusion limited binary colloidal system. *The Journal of Chemical Physics* **2017**, *147*, 054904.
